# Predictive Models of Life Span in Old Female Mice Based on Immune, Redox, and Behavioral Parameters

**DOI:** 10.3390/ijms25084203

**Published:** 2024-04-10

**Authors:** Judith Félix, Irene Martínez de Toda, Estefanía Díaz-Del Cerro, Iris Sánchez-Del Pozo, Mónica De la Fuente

**Affiliations:** 1 Animal Physiology Unit, Department of Genetics, Physiology and Microbiology, Faculty of Biological Sciences, Complutense University of Madrid, 28040 Madrid, Spain; jufelix@ucm.es (J.F.); estedi01@ucm.es (E.D.-D.C.); irissa02@ucm.es (I.S.-D.P.); mondelaf@bio.ucm.es (M.D.l.F.); 2 Institute of Investigation Hospital 12 Octubre (Imas12), 28041 Madrid, Spain

**Keywords:** aging, life span, immune system, oxidative stress, behavior, mathematical models

## Abstract

The development of mathematical models capable of predicting the lifespan of animals is growing. However, there are no studies that compare the predictive power of different sets of parameters depending on the age of the animals. The aim of the present study is to test whether mathematical models for life span prediction developed in adult female mice based on immune, redox, and behavioral parameters can predict life span in old animals and to develop new models in old mice. For this purpose, 29 variables, including parameters of immune function, redox state, and behavioral ones, were evaluated in old female Swiss mice (80 ± 4 weeks). Life span was registered when they died naturally. Firstly, we observed that the models developed in adults were not able to accurately predict the life span of old mice. Therefore, the immunity (adjusted R^2^ = 73.6%), redox (adjusted R^2^ = 46.5%), immunity-redox (adjusted R^2^ = 96.4%), and behavioral (adjusted R^2^ = 67.9%) models were developed in old age. Finally, the models were validated in another batch of mice. The developed models in old mice show certain similarities to those in adults but include different immune, redox, and behavioral markers, which highlights the importance of age in the prediction of life span.

## 1. Introduction

Nowadays, there is an increasing interest in developing mathematical models for predicting life span or the rate of aging, as these are very useful tools for understanding how an individual is aging or the effect that a specific intervention could have on this process. However, while it has been possible to develop different models that allow us to know the rate of aging in humans [1,2], developing models of life span in humans is a complex task given their long life span. For this reason, most authors seeking to develop this type of mathematical model use animals such as mice, whose average life span is two years, thus facilitating this type of study [3].

To develop these models, it is first necessary to identify different markers that indicate how an individual is aging and that are directly related to their life span [4]. Although many types of aging markers have been proposed in animals and have been used for life span prediction, most of them focus on characteristics that are consequences and not causes of this process, such as low body weight, different T-cell subpopulations, serum hormone levels, or cataract score, among others [5,6,7,8,9,10]. This is why it is necessary to develop life span prediction models based on markers that are known to be truly involved in the aging process.

Accordingly, the immune system has been proposed as an excellent marker that indicates how an individual is aging [11]. In fact, different parameters of immune function have been proposed as markers of biological age, i.e., the rate at which an individual is aging, and different mathematical models have been developed based on them [3,12]. Among these parameters, the functional capacity of phagocytes, such as their chemotaxis and phagocytic capacity, and of lymphocytes, such as their chemotaxis and proliferative response ability under basal or mitogen-stimulated conditions, as well as their natural killer antitumor capacity, have been used [12,13]. In addition, given the close relationship between the immune system and the redox state of individuals, different redox parameters have also been proposed as good markers of the aging process, such as glutathione reductase and peroxidase activities (GR, GPx), the concentration of oxidized (GSSG) and reduced (GSH) glutathione, and the GSSG/GSH ratio, as well as lipid peroxidation (concentration of thiobarbituric acid reacting substances (TBARs)) [4,12]. Among the mathematical models developed using these parameters, we can find the immunity clock developed both in humans and mice, and the redox clock developed only in mice [11,12]. These models allow us to know the biological age or rate at which each subject is aging at different stages of their lives and, likewise, to check whether different interventions in their lifestyle reduce or accelerate the rate at which they are aging. Furthermore, given the short life span of mice, the immunity clock and redox clock developed in mice demonstrated for the first time that a decrease in biological age is associated with an increase in longevity and vice versa [12]. However, although these models are very useful in the field of aging research, they only allow us to know at which rate an individual is aging, but not what his or her life span will be. Different mathematical models have been developed in adult mice based on different immune and redox parameters, including those mentioned above [3], to predict this remaining life span. In addition, behavioral parameters have also been proposed as markers of the rate of aging, given that they reflect nervous system performance, and a behavioral model based on exploratory and anxiety-like behaviors has also been developed in mice [3,14]. However, these predictive models of life span have only been developed in adult animals. Therefore, it is not known if these models can be applied to old animals since it is known that aging is accompanied by the functional deterioration of the regulatory systems, the nervous, immune, and endocrine systems, due to the establishment of chronic oxidative and inflammatory stress [11]. In fact, we showed in a previous study that certain immune and redox parameters can be pro-longevity or anti-longevity depending on the age window studied [4].

Therefore, the aim of the present study is to test whether the models developed in adults predict life span in old animals and to develop new mathematical models predictive of life span in old mice, including behavioral, immune, and redox state parameters.

## 2. Results

First, the mathematical models developed in adult mice [4] were applied to a set of old mice (N = 50). This resulted in a Pearson’s correlation coefficient of −0.247 (*p* = 0.008) for the immunity model, a Pearson’s correlation coefficient of −0.258 (*p* = 0.006) for the redox model, a Pearson’s correlation coefficient of −0.249 (*p* = 0.007) for the immunity-redox model, and finally, a Pearson’s correlation coefficient of 0.221 (*p* = 0.096) for the behavioral model.

Even though we found a statistically significant correlation when applying the immunity and redox models for life span prediction to old animals, the correlation was negative, showing that the mathematical models developed in adults are not optimal for life span prediction of old mice. Thus, we decided to construct new models based on the different sets of variables (immune, redox, and behavioral) quantified in mice in old age. To develop the life span prediction models in old age, a stepwise forward methodology was followed, which first selects the variable that most explains the dependent variable, then the next one, and so on. The criteria for variable inclusion into the model are that they had a *p* < 0.05.

For the construction of the immunity model, multiple linear regression (MLR) analysis was performed using the observed life span as the dependent variable (all mice used for data collection showed a life span between 85 and 128 weeks) and 10 parameters of immune function assessed in peritoneal leukocytes from 150 mice as independent variables: natural killer activity, macrophage phagocytic index and efficacy, macrophage and lymphocyte chemotaxis, proliferation capacity of lymphocytes without stimuli and in response to lipopolysaccharide and concanavalin A, as well as the percentage of stimulation of lymphoproliferation with both mitogens. The sequential phases of model construction are detailed in Table 1.

In the first step, the percentage of stimulation with concanavalin A was selected, then the lymphocyte chemotaxis, thirdly, the phagocytic index, and, finally, the natural killer activity. The remaining variables were excluded from the model due to their failure to meet the inclusion criteria. By incorporating the four variables, the model attained an adjusted coefficient of determination (R^2^) of 73.6% and exhibited a standard deviation of 6.31 weeks in predicting life span.

**Table 1:** Immunity model construction through the stepwise forward method. Predicted remaining life span = β_0_ − β_1_ × percentage of stimulation with ConA − β_2_ × lymphocyte chemotaxis − β_3_ × phagocytic index + β_4_ × natural killer activity.

	Model 1	Model 2	Model 3	Model 4
Constant (β_0_)	121.345 (7.683)	120.638 (6.708)	124.371 (6.261)	109.714 (7.101)
Percentage of stimulation with ConA (β_1_)	−0.222 ** (0.072)	−0.167 * (0.067)	−0.137 * (0.061)	−0.109 * (0.050)
Lymphocyte Chemotaxis (β_2_)		−0.039 * (0.016)	−0.041 ** (0.014)	−0.043 ** (0.012)
Phagocytic index (β_3_)			−0.008 * (0.004)	−0.009 ** (0.003)
Natural killer activity (β_4_)				0.325 ** (0.111)
R^2^	37.3%	55.3%	66.4%	79.8%
Adjusted R^2^	33.4%	49.3%	59.2%	73.6%

Each value shows the estimated coefficient and the standard error for each coefficient is shown in brackets. * *p* < 0.05, ** *p* < 0.01.

To develop the redox model, observed life span was used as the dependent variable, while the independent variables were glutathione reductase activity (GR), glutathione peroxidase activity (GPx), the concentration of oxidized (GSSG) and reduced (GSH) glutathione, the GSSG/GSH ratio, and the concentration of TBARs assessed in peritoneal leukocytes from 150 mice.

Table 2 shows the steps for the redox model’s construction. The first selected variable was the glutathione reductase activity (GR), and the second one was the glutathione peroxidase activity (GPx). No more variables were included as they did not fit the inclusion criteria. The model presented an adjusted R^2^ of 46.5% with these two variables and a standard error of 13.77 weeks.

We aimed to examine whether integrating both sets of parameters, namely immune and redox variables, into a unified model would enhance the precision of life span prediction. For this, all parameters evaluated in 150 mice and used for the construction of the immunity and redox models were included as independent variables, and the observed life span was used as the dependent variable. Table 3 shows the steps of model construction.

In the first step, the phagocytic index was selected; in the second, the lymphocyte chemotaxis; in the third, the concentration of TBARs; in the fourth, the GSSG/GSH ratio; and finally, in the fifth, the natural killer activity. With the inclusion of the five variables, the model reached an adjusted R^2^ of 96.4% and a standard error of 2.60 weeks for life span prediction.

**Table 3:** Immunity-redox model construction through stepwise forward method. Predicted remaining life span = β_0_ − β_1_ × phagocytic index − β_2_ × lymphocyte chemotaxis + β_3_ × TBAR concentration + β_4_ × GSSG/GSH ratio + β_5_ × natural killer activity.

	Model 1	Model 2	Model 3	Model 4	Model 5
Constant (β_0_)	113.326 (6.206)	122.067 (5.270)	112.827 (5.209)	106.777 (3.872)	98.269 (4.213)
Phagocytic index (β_1_)	−0.016 * (0.006)	−0.014 ** (0.004)	−0.012 ** (0.003)	−0.01 ** (0.002)	−0.010 ** (0.002)
Lymphocyte Chemotaxis (β_2_)		−0.092 ** (0.030)	−0.131 ** (0.027)	−0.126 *** (0.017)	−0.108 *** (0.014)
TBAR concentration (β_3_)			11.080 * (4.090)	10.283 ** (2.675)	8.378 ** (2.020)
GSSG/GSH ratio (β_4_)				3.410 ** (1.052)	3.782 ** (0.756)
Natural killer activity (β_5_)					0.180 * (0.068)
R^2^	46.8%	75.7%	88.1%	95.7%	98.2%
Adjusted R^2^	40.8%	69.6%	83.1%	92.8%	96.4%

Each value shows the estimated coefficient and the standard error for each coefficient is shown in brackets. * *p* < 0.05, ** *p* < 0.01, *** *p* < 0.001.

The construction of the behavioral model is shown in Table 4. For this model, we used another set of 60 mice since we could not evaluate the behavioral response in the set of mice used for the development of the immunity, redox, and immunity-redox models. For the development of the behavioral model, observed life span was placed as the dependent variable, and as independent variables, different behavioral parameters were studied in the elevated plus maze (EPM) and the hole-board (HB) tests, as specified in Section 4.

The first variable introduced was the time spent in the closed arms in the EPM; the second one, the number of head-dippings in the EPM; the third one, the time spent in the open arms in the EPM; the fourth one, the time to exit from the external zone in the hole-board test (HB); and finally, in the fifth, the number of groomings in the HB. With the inclusion of the five variables, the model reached an adjusted R^2^ of 67.9% and a standard error of 7.39 weeks for life span prediction.

**Table 4:** Behavioral model construction through the stepwise forward method. Predicted remaining life span = β_0_ − β_1_ × time in closed arms (EPM) + β_2_ × head-dippings (EPM) − β_3_ × time in open arms (EPM) − β_4_ × time to exit from the external zone (HB) + β_5_ × number of groomings (HB).

	Model 1	Model 2	Model 3	Model 4	Model 5
Constant (β_0_)	124.971 (5.455)	107.317 (7.601)	125.223 (8.756)	126.333 (8.363)	124.706 (8.018)
Time in closed arms (EPM) (β_1_)	−0.205 *** (0.035)	−0.145 *** (0.038)	−0.217 *** (0.040)	−0.214 *** (0.038)	−0.226 *** (0.037)
Head-dippings (EPM) (β_2_)		0.412 ** (0.134)	0.464 *** (0.122)	0.479 *** (0.116)	0.571 *** (0.119)
Time in open arms (EPM) (β_3_)			−0.141 ** (0.043)	−0.140 *** (0.041)	−0.164 *** (0.041)
Time to exit from the external zone (HB) (β_4_)				−2.053 * (0.909)	−2.708 ** (0.917)
Number of groomings (HB) (β_5_)					0.909 * (0.411)
R^2^	44.1%	54.4%	63.9%	68.0%	71.5%
Adjusted R^2^	42.8%	52.2%	61.2%	64.8%	67.9%

Each value shows the estimated coefficient and the standard error for each coefficient is shown in brackets. * *p* < 0.05, ** *p* < 0.01, *** *p* < 0.001.

**Figure 1:**
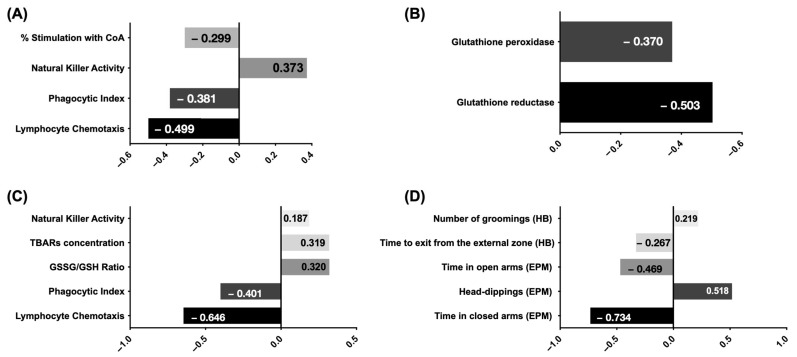
Relative contribution of each variable towards life span prediction in (**A**) immunity model; (**B**) redox model; (**C**) immunity-redox model; (**D**) behavioral model. The contribution of each variable is expressed as its standardized beta coefficient.

The standardized beta coefficients were computed for each model variable to determine each variable’s unique contribution across various adjusted models, as depicted in Figure 1. Standardization of coefficients is commonly employed to discern the relative influence of independent variables on the dependent variable in multiple regression analysis, particularly when the variables are measured in disparate units, as in the present study.

**Figure 2:**
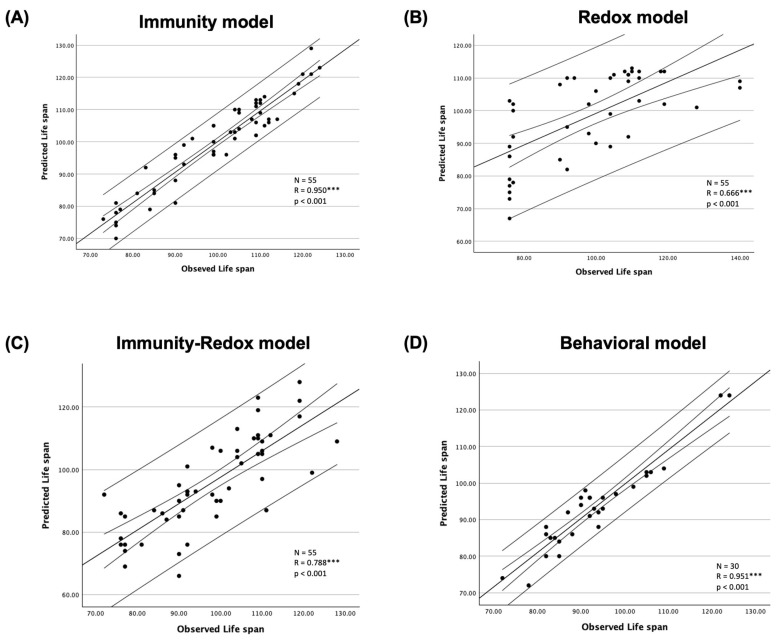
Validation of the (**A**) immunity model, (**B**) redox model, and (**C**) immunity-redox model was conducted in an additional cohort comprising 55 female mice, whereas validation of the (**D**) behavioral model was performed in a distinct cohort consisting of 30 female mice. The narrower lines represent the mean confidence interval, while the wider lines denote the individual confidence interval, both set at a 95% confidence level. Dots represent individual predicted versus observed life span for each mouse. *** *p* < 0.001.

The subsequent phase involved the validation of model predictability across distinct cohorts of aged Swiss female mice. Validation encompassed the immunity, redox, and immunity-redox models in one cohort consisting of 55 mice, while the behavioral model was validated in a separate cohort comprising 30 mice (Figure 2). The immunity model yielded a Pearson correlation coefficient of 0.950 (*p* < 0.001) between predicted and observed life span. Similarly, the redox model demonstrated a Pearson correlation coefficient of 0.666 (*p* < 0.001), while the immunity-redox model exhibited a coefficient of 0.788 (*p* < 0.001). Furthermore, the behavioral model displayed a Pearson correlation coefficient of 0.951 (*p* < 0.001) between predicted and observed life span.

## 3. Discussion

The development of different mathematical models capable of predicting how long an individual will live is growing. However, this is the first study that highlights the importance of the age of individuals in the development of these models.

Aging can be defined as the age-related deterioration of the three homeostatic systems, namely the nervous, endocrine, and immune systems, and their communication, which preserve one organism’s health [11]. It has been proposed that the age-related impairment of these systems is due to the establishment of a chronic oxidative stress situation. Accordingly, different life span prediction models have been developed based on immune, redox, and behavioral parameters evaluated in mice at the adult age [3,14].

Nevertheless, the parameters selected in those predictive models are known to experience age-related changes, and previous studies have shown that some immune and redox markers can be pro-longevity at the adult age and anti-longevity in old age or vice versa [4]. Thus, the first objective of the present work was to test whether mathematical models of life span prediction developed in mice during adulthood are capable of accurately predicting the life span of individuals when they reach old age. It was found that the models developed in adulthood fit poorly to mice of this age and, therefore, are not suitable for accurately predicting their remaining life span. It was, therefore, decided to develop new predictive models based on the same sets of variables in old mice.

The immunity model developed for old animals includes the following parameters: percentage of stimulation with concanavalin A, lymphocyte chemotaxis, phagocytic index, and natural killer activity, and it can predict 73.6% of the life span of individuals with an error of 6.36 weeks. We found similarities with respect to the model developed in adults since both formulas include the phagocytic index and the capacity of stimulation with concanavalin A, reinforcing their important role in the aging process independent of the age window studied [3]. However, while the model developed in adults includes immune functions more related to the innate branch (the aforementioned parameters and macrophage chemotaxis capacity), the model developed in old mice includes immune functions more related to the adaptive branch. The fact that macrophage functions are the strongest predictors of life span in adulthood agrees with the hypothesis of phagocytes being the cell type that contributes the most to the aging process, given their unique ability to produce pro-inflammatory cytokines and reactive oxygen species [11,13], since it is at this adult age when the aging process starts. Strikingly, our results suggest that the functional capacity of lymphocytes becomes a strong determinant of life span in old age. Accordingly, it has been described that with aging, there is a more pronounced deterioration of the adaptive immunity branch, mainly affecting lymphocytes, so it would make sense that it is in old age when these immune parameters have greater predictive power [4,13,15,16].

In the redox model developed in old animals, glutathione reductase and glutathione peroxidase activities are included, being able to predict the life span of the animal in 46.5% of cases and with an error of 13.77 weeks. This model described in old mice is very different from the one developed in adult mice, where the variables included were reduced glutathione concentration, malondialdehyde concentration, and glutathione peroxidase activity [3]. One fact that can be observed by comparing both models is that while the model described in adult animals is capable of predicting life span with an accuracy of 84.4% [3], the one developed in old animals only explains 46.5% of the observed variance in life span. This could be due to the fact that during adulthood, the organism tends to maintain a balance between oxidant and antioxidant compounds, whereas, in old age, a chronic oxidative state has already been established, i.e., the redox balance has been broken, favoring oxidant compounds [11]. Thus, it is logical to think that these redox parameters do not allow for discrimination between the different remaining life spans between individuals in old age. This would explain the lower predictive capacity of the redox model developed in old mice versus the one developed in adults or even versus the immunity model developed in old mice since the immune system shows a great heterogeneity among individuals once old age is reached [4,12].

Subsequently, it was sought to develop an immunity-redox model in old animals like the one previously described in adults [3]. The model developed in old age included the following variables: phagocytic index, lymphocyte chemotaxis, TBAR concentration, GSSG/GSH ratio, and natural killer activity. This model can predict life span with a 96.4% accuracy and with an error of 2.60 weeks and includes some different variables from the one developed in adults, which included reduced glutathione concentration, malondialdehyde concentration, phagocytic index, glutathione peroxidase, percentage of stimulation with ConA, and macrophage chemotaxis [3]. Again, it can be observed that in the model developed in adults, the parameters most capable of predicting life span are those related to the redox state, while in the model developed in old mice, the strongest parameters for life span prediction are related to immune function, being the most important ones the lymphocyte chemotaxis and the phagocytic index.

Finally, in the behavioral model developed in old mice, the parameters included were the time spent in closed arms (EPM), the number of head-dippings (EPM), the time in open arms (EPM), the time to exit from the external zone (HB) and the number of groomings (HB). With these parameters, this model predicts life span with an accuracy of 67.9% and with an error of 7.39 weeks. This model includes more parameters than the one developed in adult animals, where only internal locomotion (HB) and time in open arms (EPM) were included [3]. It is noteworthy that both behavioral models include parameters that warn of the individual’s anxiety levels [17,18,19,20]. The reason why, at old age, more parameters are needed to reach a similar life span prediction accuracy could be dependent on the fact that with aging, there is an increase in anxiety-like behaviors in mice [11,21]. Thus, it could be easier to discriminate between anxious and non-anxious mice in adulthood. However, in aged mice, due to their higher anxiety-like responses [11], more parameters are necessary to differentiate between the different anxiety behaviors to establish a relationship with longevity.

It is worth mentioning that if we observe the predictive capacity of the different models developed in old animals, we find some similarities with respect to the predictive capacity of those developed in adults. Independently of age, the model with the highest predictive capacity is the immunity-redox model. This fact reinforces one of the theories proposed to explain the aging process, the oxidation–inflammation theory of aging, which states that aging unfolds because of the establishment of a chronic oxidative stress situation that leads to low-grade chronic inflammation throughout the immune system’s activity [11].

Overall, the results of our study highlight the need to develop mathematical models for life span prediction at different ages, given the fact that the relationship of some parameters towards life span can be distinct or even contrary depending on the age point studied. Likewise, it is worth mentioning that the main limitation of this study is that it has been performed using exclusively females, given the facility to study their immune system as opposed to males, which are more aggressive, and this leads to lesions that would affect the studied parameters [22]. In fact, it is known that there are sex differences, such as higher longevity in females, as well as differences in the immune, inflammatory, and redox state of females and males [14]. Therefore, the parameters able to predict longevity in old male mice could be different from those described in old female mice. In this way, previous studies have already shown that mathematical models developed in female mice when applied to male mice are not valid; therefore, it is necessary to develop new models for this sex [14]. Nevertheless, knowing the differences that exist due to sex [14], the developed mathematical models in old female mice should be applied to male mice to ascertain if they can be valid or if the parameters for life span prediction in old age are sex-dependent. Moreover, although in this study the investigated markers of immune function and redox state were those that were previously proposed by our research group as markers of longevity [3,4,13], it would also be interesting for future studies to include other markers of immune function, inflammation, and oxidative stress, especially those that allow a better characterization of the redox profile, such as superoxide dismutase, catalase, and thioredoxin, which may also be related to the life span of the animals.

Finally, it should be mentioned that the development of these new life span prediction models in old mice can contribute through shedding light on the mechanisms that govern longevity in old age by the identification of the strongest determinants of life span at this age. Moreover, these life span prediction models would allow testing the effect that a certain intervention during old age, such as supplementation with antioxidants, probiotics, or exposure to a positive social environment, will have an impact on mice life span, without the need to wait until all mice die.

## 4. Materials and Methods

### 4.1. Animals and Experimental Design

For this study, ex-reproductive female Swiss mice (Janvier, Tancon, France) were used. We used the Swiss strain due to its higher genetic heterogeneity than that of inbred strains, which could facilitate the extrapolation of the results to humans [23]. Female mice were used for this study because male mice show aggressive and dominant behavior when caged together, and it has been shown that social isolation causes alterations in neuroimmunoendocrine communication, affecting the aging rate [22]. We used ex-reproductive females, given that it better replicates the most frequent situation in human aging. In addition, in ex-reproductive female mice, the cyclicity of the estrous cycle ceases (maintaining the diestrus phase), and because of that, the hormones do not affect the parameters studied in the present work [24].

They were housed 6 per cage and maintained with ad libitum access to food and tap water under light (12/12 h reversed light/dark cycle; lights off at 8:00 a.m.) to avoid circadian interferences. Temperature (22 ± 2 °C) and humidity (50–60%) were also controlled. The diet was in accordance with the American Institute of Nutrition recommendations for laboratory animals (A04 diet from Panlab S.L., Barcelona, Spain). Composition: water, 12%; protein, 18%; fat, 3%; fiber, 4.3%; starch, 35%; total sugar, 3.5%; ash, 8%; phosphorus, 0.65%; sodium chloride, 0.6%; calcium, 1.28%; vitamin A, 15,000 IU/kg; cholecalciferol, 2000 IU/kg; vitamin E, 15 mg; energy value, 2900 Cal/kg). Experiments were performed during the dark phase of the cycle (8:00–12:00 h). It was decided to perform the experiments at this time because the animals have biological clocks. These clocks are measured by different stimuli, including the light–dark cycle [25]. Thus, rodents show their active phase during the night, this being the time when they behave in a more natural way and when their immune cells function works in the most optimal way [26,27,28].

The protocol was approved by the Experimental Animal Committee of Complutense University of Madrid (Spain) (PROEX 224.0/21). Animals were treated according to the European Community Council Directives ECC/566/2015 guidelines.

To test if the models developed in adult mice predict life span in old mice, a group of 50 Swiss females of 80 ± 4 weeks old was used.

For the development of the mathematical models (immunity, redox and immunity-redox) one group of mice (N = 150) of 80 ± 4 weeks old was used for immune function and redox data collection. For this purpose, peritoneal leukocytes were extracted, and several immunological parameters were analyzed including the following: phagocytic index, phagocytic efficiency, macrophage chemotactic index, lymphocyte chemotactic index, natural killer activity, basal lymphoproliferative response, lymphoproliferative response to concanavalin A (ConA), lymphoproliferative response to lipopolysaccharide (LPS), percentage of stimulation with ConA, and percentage of stimulation with LPS to evaluate the state of the immune system. Different redox state parameters were also analyzed: glutathione reductase and peroxidase activities, oxidized and reduced glutathione concentration, and thiobarbituric acid reactive substances (TBARs) to assess lipid peroxidation. For the behavioral model, a group of 60 mice of 80 ± 4 weeks old was used for performing the elevated plus maze and the hole-board test, where several parameters were analyzed as described below.

To validate the immunity, redox, and immunity-redox models, another group of mice (N = 55) of 80 ± 4 weeks was used, from which peritoneal leukocytes were extracted, and the abovementioned immune functions and redox state parameters were assessed. To validate the behavioral model, another group (N = 30) of 80 ± 4 weeks was used.

Animals were monitored individually along the aging process, and each individual’s achieved life span was recorded.

### 4.2. Behavioral Tests

Behavioral testing took place for four consecutive days. Tests and sequence were chosen based on previous reports [29]. On the first day, animals were subjected to the hole-board test, and on the second day, they were exposed to the elevated plus maze. Tests were carried out under red light with a white light lamp (20 W) and were started by placing the animals in the area of the apparatus considered most behaviorally neutral so that the mouse was not artificially induced to perform a significant pattern [30]. The apparatuses were cleaned to avoid possible olfactory interferences with 70% ethanol between animals.

#### 4.2.1. Hole-Board Test

To analyze the non-goal-directed behavior (evaluated by horizontal and vertical activity) as well as goal-directed behavior (evaluated by the number and time of head-dipping), mice performed the hole-board test. The device consists of a box (60 cm × 60 cm × 45 cm) divided into 36 squares, with four equidistant holes in the inner zone. The external zone is the 20 squares nearest the walls, and the remaining squares are the inner zone. In each hole, plastic objects were placed to attract the animal’s attention and drive their goal-directed behavior. The duration of the test was 5 min [3], and during this time, parameters for non-goal-directed and goal-directed behavior were recorded. For non-goal-directed behavior, we evaluated the time to exit from the external zones, the total, external, middle, and inner locomotion (the number of crossed squares in each area), and the percentage of all of them (the number of crossed squares in each area divided by the total locomotion). All these parameters were considered horizontal activity. Regarding vertical activity, the number of wall and central rearings and the time(s) of each rearing were recorded. Finally, for goal-directed behavior, the total number of head-dippings and the time(s) of each head-dipping were evaluated. Other behaviors, such as grooming and freezing (number and time in seconds), were also considered.

#### 4.2.2. Elevated Plus Maze

Anxiety levels were evaluated by the elevated plus maze test [31]. The device consists of two open arms (45 cm × 10 cm) and two closed arms (5 cm × 10 cm × 50 cm) that extend from a central platform (10 cm × 10 cm), elevated 65 cm above the floor. The duration of the test was 5 min. Mice were placed on the central platform facing a closed arm, and the total number of entries in open and closed arms, time spent (s) in the central platform, and in open and closed arms were evaluated. Other behaviors such as head-dippings (number), grooming, and rearing (number and time in seconds) were also recorded.

### 4.3. Extraction of Peritoneal Leukocytes

The extraction of peritoneal leukocytes was performed between 8:00 and 10:00 a.m. to minimize circadian variations in the studied immune parameters following a protocol already described [13]. Without the use of anesthesia, mice were immobilized by taking a fold of the neck and the entire dorsal area of the animal, and the abdominal area was cleaned with 70% ethanol. Subsequently, 3 mL of sterile Hank’s solution was injected intraperitoneally, and abdominal massage was performed. Then, 80% of the volume injected with the needle used for the injection of Hank’s solution was recovered. The leukocytes of the peritoneal suspensions obtained were identified by their morphology (macrophages or lymphocytes) and quantified (number of cells/mL) in a Neubauer chamber using light microscopy (×40). Cell viability was assessed by trypan blue exclusion test, and only suspensions with a viability greater than 95% were used.

### 4.4. Immune Function Parameters

#### 4.4.1. Macrophage and Lymphocyte Chemotaxis

This was performed following the technique described by Boyden (1962) and modified by De la Fuente’s group [13]. It was evaluated by taking 300 μL of the peritoneal suspension containing macrophages or lymphocytes, adjusted to 500,000 cells/mL of Hank’s solution, that was deposited in the upper compartment of a Boyden chamber separated by a nitrocellulose filter with pores of 3 μm in diameter. Then, 400 μL of formylated peptide (N-formyl-methionyl-leucyl-phenylalanine), a chemoattractant agent, was added in the lower compartment of the Boyden chamber to induce chemotaxis. After 3 h of incubation at 37 °C and 5% CO_2_, the filter-bound cells were fixed with 50% methanol and 75% ethanol and stained with azur–eosin (GIEMSA, PANREAC, Barcelona, Spain). Finally, the number of macrophages or lymphocytes that had passed through the filter (found on the underside) was counted by optical microscopy, and the chemotactic index (C.I.) was calculated.

#### 4.4.2. Macrophage Phagocytosis

The technique described by De la Fuente [13] was used. A total of 200 μL of peritoneal suspension adjusted to 500,000 macrophages/mL was incubated on migration inhibition factor (MIF) plates for 30 min. The adherent monolayer was washed with phosphate-buffered saline (PBS) at 37 °C and 20 μL of latex beads (1.09 μm diluted 1% PBS, Sigma-Aldrich, St. Louis, MO, USA) was added. After 30 min incubation, the sample was fixed with 50% methanol and stained with azur–eosin blue (GIEMSA, PANREAC). The number of particles per 100 macrophages (phagocytic index) and the percentage of macrophages that ingested at least one particle (phagocytic efficiency) were determined by optical microscopy (100×).

#### 4.4.3. Natural Killer Activity

An enzymatic colorimetric kit (Cytotox 96™ Promega, Madison, WI, USA; Boehringer Ingelheim, Ingelheim, Germany) based on the determination of lactate dehydrogenase (LDH) released by cytolysis of target cells (tumor cells) was performed using tetrazolium salts. The peritoneal suspension (adjusted to 10^6^ cells/mL culture medium) was added to 96-well U-bottom culture plates with target cells (murine YAC-1 tumor cells) in a 10:1 ratio. After 4 h of incubation, LDH was measured by the addition of the enzyme substrate at an absorbance of 490 nm.

The formula to calculate this function is:

$$Lysis\;\%=\frac{Problem\;lysis-Effector\;cells\;spontaneous\;lysis-Tumor\;cells\;spontaneous\;lysis}{Tumor\;cells\;total\;lysis-Tumor\;cells\;spontaneous\;lysis}\times 100$$

where problem lysis of effector cells refers to the mean of well absorbances where the observed lysis is caused by the action of effector cells (peritoneal leukocytes) on target cells (YAC-1). Spontaneous lysis of effector cells is lysis due to the death of peritoneal leukocytes naturally during the process. The total lysis of target cells is the mean absorbance of those wells where all tumor cells have been lysed by the addition of a lysis solution. Finally, the spontaneous lysis of tumor cells is the mean of the lysis absorbances due to natural tumor cell death during the process [13].

#### 4.4.4. Lymphoproliferative Response

Peritoneal leukocyte suspensions adjusted to 10⁶ lymphocytes/mL of complete RPMI (supplemented with gentamicin (1 mg/mL) and 10% fetal bovine serum (Gibco, Billings, MT, USA), previously heat decomplemented for 30 min at 56 °C), were incorporated in aliquots of 200 μL/well, in 96-well plates. The following were added to the wells: 20 μL of RPMI medium in the case of the basal condition and 20 μL of concanavalin A (ConA) or lipopolysaccharide (LPS) (1 μg/mL) for the mitogen response. After 48 h of incubation, tritiated thymidine was added, followed by another 24 h of incubation. Cells were fixed on a filter by an automated machine, and thymidine tritiated was measured in a beta counter. The results were expressed as counts per minute (c.p.m.) for basal and stimulated conditions. In addition, the percentage of stimulation was assessed, that is, mitogen-stimulated lymphoproliferation divided by basal lymphoproliferation ×100 into cases of mitogen-stimulated proliferative response [13].

### 4.5. Oxidative Stress Parameters

#### 4.5.1. Glutathione Reductase Activity (GR)

The glutathione reductase activity was analyzed following a method previously described [32]. Peritoneal leukocytes (10^6^ cells/mL) were resuspended in oxygen-free phosphate buffer (pH 7.4, 50 mM with 6.3 nM EDTA). They were then sonicated, and after centrifugation at 3200× *g* at 4 °C for 20 min, supernatants were collected. GSSG (80 mM) was used as a substrate and following the decrease in absorbance by oxidation of NADPH at 340 nm for 4 min, the activity was calculated. Results are expressed as milliunits (mU) of GR per milligram of protein (mU GR/mg protein).

#### 4.5.2. Glutathione Peroxidase Activity (GPx)

The glutathione peroxidase activity was assayed using a previously described method with some modifications [4]. Peritoneal leukocytes (10^6^ cells/mL) were resuspended in oxygen-free phosphate buffer (pH 7.4, 50 mM). They were then sonicated, and after centrifugation at 3200× *g* at 4 °C for 20 min, the supernatants were collected. Cumene hydroperoxide (cumene-OOH; Sigma) was used as substrate. The activity was quantified by measuring the decrease in absorbance at 340 nm by oxidation of NADPH in the presence of excess glutathione reductase (GR) for 5 min. Results are expressed as milliunits (mU) of GPx activity per milligram of protein (mU GPx/mg protein).

#### 4.5.3. Oxidized (GSSG) and Reduced (GSH) Glutathione Concentrations

GSSG and GSH were measured following a fluorometric assay [33]. This method is based on the ability of glutathione (both GSSG and GSH) to react with o-phthalaldehyde (OPT) at pH 12 and pH 8, respectively, forming a fluorescent compound. Peritoneal leukocytes (10^6^ cells/mL) were resuspended in phosphate buffer (pH 8, 50 mM, 0.1 M EDTA). Samples were then sonicated, and 5 μL of HClO4 (60%) was added and centrifuged for 20 min at 3200× *g*. Then, 10 μL of the supernatants was dispensed into dark-bottomed 96-well plates (one for GSSG and one for GSH). For GSSG quantification, 5 μL of N-ethylmaleimide (NEM, 0.04 M) was added to the samples to prevent oxidation of existing GSH and incubated for 30 min. Subsequently, 190 μL of NaOH and 20 μL of OPT were added to each well, and fluorescence was measured at 420 nm. In the case of GSH, 10 μL of the supernatants was added to the opaque plates, followed by adding 190 μL of 50 mM phosphate buffer at pH 8 and 20 μL of OPT, and fluorescence was measured at 420 nm. Results are expressed as nanomoles (nmol) of GSSG or GSH per milligram of protein (nmol GSSG/mg protein; nmol GSH/mg protein). In addition, the GSSG/GSH ratio was calculated.

#### 4.5.4. Concentration of Thiobarbituric Acid Reactive Substances (TBARs)

TBARs were quantified using the commercial “Lipid Peroxidation Assay Kit” (Biovision, San Francisco, CA, USA). Peritoneal leukocytes (10^6^ cells/mL) were resuspended in 300 μL of lysis buffer (containing butylated hydroxytoluene; BHT), sonicated, and centrifuged at 13,000× *g* for 10 min. Supernatants were collected, mixed with 600 μL of thiobarbituric acid (TBA), and incubated in a 95 °C water bath for 60 min. Then, 300 μL of butanol was added and centrifuged at 1700× *g* for 15 min, allowing organic phase extraction. A total of 200 μL of this was collected and transferred to a 96-well plate, and absorbance was measured at 532 nm. Results are expressed as nanomole (nmol) TBARs per milligram protein (nmol TBARs/mg protein).

### 4.6. Protein Quantification

Protein assessment was carried out on the same supernatants collected from the analysis of the different redox parameters. Protein quantification was performed by the bicinchoninic acid (BCA) method, using the BCA kit (Merck, Darmstadt, Germany), which is based on the reduction of Cu^2+^, generating Cu^+^ ions that bind to BCA and form a colored compound that absorbs light at 562 nm. The results are expressed in milligrams of protein per milliliter (mg protein/mL).

### 4.7. Model Construction and Statistical Analysis

To develop the mathematical models for estimating the life span in mice, a multiple linear regression analysis was performed using the SPSS 29.0.2 statistical program. For the immunity model, the recorded life span of the mice was introduced as the dependent variable and as predictor variables: phagocytic index and efficacy, macrophage and lymphocyte chemotaxis, natural killer activity, basal, and concanavalin A- and lipopolysaccharide-stimulated lymphoproliferation, and concanavalin A and lipopolysaccharide stimulation percentages. For the redox model, the recorded life span of the mice was introduced as the dependent variable and as predictor variables: glutathione peroxidase activity, glutathione reductase activity, the concentration of oxidized and reduced glutathione, GSSG/GSH ratio, and concentration of TBARs. For the immunity-redox model, the recorded life span of the mice was introduced as the dependent variable, and all immunity and redox parameters mentioned above were used as predictor variables. Finally, for the behavioral model, the recorded life span of the mice was introduced as the dependent variable and as predictor variables, the external, middle, and inner locomotion and their percentages, head-dipping, grooming, central and wall rearing (number and time) in the hole-board test, and from the elevated plus maze, time and number of entries in open and closed arms, time in central platform, and number of head dippings. All parameters are recorded in Table 5.

All those models were generated through multiple linear regression following the “step forward” methodology, which first selects the variable considered most explanatory for the prediction of the independent variable and then selects and adds the rest of the variables to the model one by one if they satisfy the condition of being statistically significant once they are incorporated into the model with *p* < 0.05. The models were tested for normality of the residuals, constant variability of the residuals (homoscedasticity), and the independence of the residuals was tested to verify the Gauss–Markov hypothesis through the corresponding graphical and analytical analysis.

Subsequently, the models were validated by calculating Pearson’s correlation coefficient between the observed and predicted life span in different sets of mice using GraphPad Prism 10.1.1.

**Table 5:** Set of variables used for model construction.

Immune Function Parameters	Redox Parameters	Behavioral Parameters	
Macrophage Chemotaxis	Glutathione Reductase activity	**Hole-board test (HB)**	Time to exit from the external zone
Lymphocyte Chemotaxis	Glutathione Peroxidase activity		Total, internal and external locomotion
Phagocytic index	Reduced Glutathione (GSH)		Percentage of inner and external locomotion
Phagocytic efficacy	Oxidized Glutathione (GSSG)		Number and time of rearing
Natural killer activity	GSSG/GSH ratio		Number and time of grooming
Basal lymphoproliferation	TBAR concentration		Number and time of head-dipping
Lymphoproliferation with Concanavalin A			Number and time of freezing
Lymphoproliferation with lipopolysaccharide			
Percentage of stimulation with concanavalin A		**Elevated plus maze (EPM)**	Time in open arms
Percentage of stimulation with lipopolysaccharide			Number of entries in open arms
			Time in closed arms
			Number of entries in closed arms
			Time in central platform
			Number of head-dippings

## Figures and Tables

**Table 2:** Redox model construction through stepwise forward method. Predicted remaining life span = β_0_ − β_1_ × glutathione reductase − β_2_ × glutathione peroxidase.

	Model 1	Model 2
Constant (β_0_)	108.134 (3.745)	113.827 (3.955)
Glutathione reductase activity (GR) (β_1_)	−0.604 *** (0.137)	−0.499 *** (0.130)
Glutathione peroxidase activity (GPx) (β_2_)		−0.252 ** (0.089)
R^2^	37.1%	49.7%
Adjusted R^2^	35.2%	46.5%

Each value shows the estimated coefficient and the standard error for each coefficient is shown in brackets. ** *p* < 0.01, *** *p* < 0.001.

## Data Availability

Data will be available upon request to Irene Martínez de Toda (imtcabeza@ucm.es) or Judith Félix (jufelix@ucm.es).
